# ZCCHC13-mediated induction of human liver cancer is associated with the modulation of DNA methylation and the AKT/ERK signaling pathway

**DOI:** 10.1186/s12967-019-1852-0

**Published:** 2019-04-02

**Authors:** Zhiming Li, Zhi Li, Linjun Wang, Chen Long, Zaozao Zheng, Xuan Zhuang

**Affiliations:** 1 Institute of Reproductive Health/Family Planning Research Institute, Tongji Medical College, Huazhong University of Science and Technology, Wuhan, 430030 Hubei China; 2 School of Pharmaceutical Sciences, Xiamen University, Xiamen, 361102 Fujian China; 3 Department of Urology, The First Affiliated Hospital of Xiamen University, Xiamen, 361003 Fujian China; 4 Department of Clinical Medicine, Fujian Medical University, Fuzhou, 350108 Fujian Province China

**Keywords:** ZCCHC13, Hepatocellular carcinoma, DNA methylation

## Abstract

**Background:**

Previous studies have shown that zinc-finger CCHC-type containing 13 (ZCCHC13) is located in an imprinted gene cluster in the X-inactivation centre, but few published studies have provided evidence of its expression in cancers. The CCHC-type zinc finger motif has numerous biological activities (such as DNA binding and RNA binding) and mediates protein–protein interactions. In an effort to examine the clinical utility of ZCCHC13 in oncology, we investigated the expression of the ZCCHC13 mRNA and protein in hepatocellular carcinoma (HCC).

**Methods:**

The expression of the ZCCHC13 mRNA and protein was evaluated using real-time reverse transcriptase-PCR, Western blotting and immunochemistry. DNA methylation was measured by methylation-specific PCR and bisulfite sequencing. The role of ZCCHC13 methylation was further evaluated using the demethylating agent, 5-aza-2′-deoxycytidine. The presence of anti-ZCCHC13 antibodies was determined by an ELISA.

**Results:**

ZCCHC13 expression was frequently upregulated in human liver cancer cells and tissues. Compared with heathy individuals, sera from patients with HCC displayed a significant response to the recombinant ZCCHC13 protein. The overexpression of ZCCHC13 in HCC was attributed to DNA hypomethylation in the promoter region. Moreover, overexpression of ZCCHC13 in liver cancer cells promoted cell cycle progression by facilitating the G1-S transition, which was related to aberrant activation of the ATK/ERK/c-MYC/CDK pathway.

**Conclusions:**

Based on our findings, ZCCHC13 functions an oncogene for HCC, and DNA hypomethylation is a driving factor in carcinogenesis.

**Electronic supplementary material:**

The online version of this article (10.1186/s12967-019-1852-0) contains supplementary material, which is available to authorized users.

## Background

Hepatocellular carcinoma (HCC) is one of the most common primary malignant cancers and displays a predominant increase in incidence and mortality rates in patients with cirrhosis or chronic liver diseases. Currently, HCC is the third leading cause of cancer-related death worldwide, with more than 500,000 people affected [1]. The highest incidence of HCC occurs in countries of Asia and Africa, because the high prevalence of hepatitis B and hepatitis C predisposes people residing in these countries to the development of HCC. The optimal treatments for patients with HCC are resection or transplantation, but in the early stage, this disease is characterized by slow growth and few symptoms appear until the late stage [2]. Moreover, alpha-fetoprotein (AFP) shows a low sensitivity and the lack of other serological markers increases the difficulty in detecting and treating HCC. With the aim of removing these obstacles, many laboratories are committed to identifying and studying novel HCC markers.

The MAPK/ERK pathway is a chain of proteins that transmit a signal from a receptor on the surface of the cell to the DNA in the nucleus. Because ERK signal transduction regulates cell proliferation, migration and survival, the constitutive activation of the ERK signaling pathway in HCC is not surprising. An approximately seven-fold increase in ERK1/2 phosphorylation was detected in HCC compared with surrounding benign liver tissues [3]. Notably, crosstalk between the ERK and AKT pathways regulates cell growth and development to a greater extent than either pathway alone. An animal model has confirmed the existence of a complex interplay between the AKT and ERK pathways during hepatocarcinogenesis [4]. However, the upstream factors that trigger the AKT/ERT pathways remain largely unexplored.

DNA methylation is a heritable change involving the transfer of a methyl group onto the 5-position of the cytosine ring to form methyl cytosine. This epigenetic modification usually occurs at a CpG dinucleotide within gene promoters. Alterations in DNA methylation are one of the most widely concerned epigenetic changes in human cancers. Genome-wide evidence previously revealed abnormal methylation signatures in patients with HCC [5]. Hypermethylation of tumor suppressor genes is generally considered a remarkable feature of the cancer genome. Additionally, global hypomethylation is another known driver of carcinogenesis and is particularly associated with the activation of cancer–testis (CT) genes [6]. CT genes are a group of tumor-associated antigens expressed in human tumors but not in normal tissues, except for the testis, making them an ideal target for cancer immunotherapy. Ten percent of genes on the X-chromosome are estimated to belong to CT-X family [7]. Currently, all CT-X genes have been shown to contain methylated CpG islands in normal somatic tissues, and demethylation is thought to be an epigenetic factor that reactivates CT gene expression during carcinogenesis [8].

Microarray technology has been successfully applied to identify novel biomarkers in a variety of human diseases [9–11]. Recently, we established an integrated analysis between gene expression and DNA methylation using testis samples from patients with non-obstructive azoospermia (NOA) [12]. Downregulation of Zinc finger CCHC-type containing 13 (ZCCHC13) was shown to be associated with spermatogenesis failure in patients with NOA. Available data collections revealed that the coding sequence of ZCCHC13 was highly conserved in mammalian species. ZCCHC13 is mapped to the Xq13.2 region of the human X chromosome, which is enriched in CT genes [13]. In 2005, Hahn and colleagues identified human-specific mutations in ZCCHC13, providing researchers the opportunity to study the acquisition of human-specific traits [14]. However, evidence of its carcinogenic effects on any cancers has not been published. In this study, we observed ZCCHC13 overexpression in liver cancer cells. Then, we tested the hypothesis that ZCCHC13 overexpression might be related to global DNA hypomethylation and promote hepatocellular carcinoma (HCC).

## Materials and methods

### Tissues and sera

Sixty-one patients with HCC and 38 healthy controls were examined (Additional file 1: Tables S1, S2). Tissue specimens (including tumor and paired noncancerous tissues) and serum samples were collected after obtaining written informed consent from patients undergoing surgery at the First Affiliated Hospital of Xiamen University under the protocol approved by Institutional Ethics Committee. Tumor tissues and sera were immediately snap frozen and stored at − 80 °C.

### Cell culture and drug treatments

Four human lung cancer cell lines, seven human liver cancer cell lines, seven human stomach cancer cell lines, and other cancer cell lines (HeLa, MCF7, and PC3), were used in the immunoblot assay. All cancer cell lines were cultured in the recommended medium under standard conditions. HCC cells were incubated with four doses (1, 10, 22, or 44 µM) of 5-aza-2′-deoxycytidine (5-Aza) (Sigma, St. Louis, MO), a demethylation treatment, for the indicated times. After 24 h and 48 h of incubation, mRNA and protein levels were analyzed. The medium containing 5-Aza was replaced every 24 h.

### Quantitative reverse transcription (RT)-PCR

Total RNA was extracted from human cell lines using the TRIzol Reagent according to the manufacturer’s protocol. The concentration and quality of RNA were measured and confirmed using a NanoDrop ND-1000 (Peqlab, Erlangen, Germany) and electrophoresis, respectively. The cDNA templates were synthesized using Quanti-Tect Reverse Transcription Kit (Qiagen, Hilden, Germany). Human ZCCHC13 primers were 5′-ACGAGAGAGACGCCAACACTG-3′ (forward) and 5′-CGCATCGGTAACACTTGACCT-3′ (reverse). GAPDH expression was examined as an internal control [15]. RT-PCR was performed as follows: 94 °C for 30 s; 40 cycles of 94 °C for 5 s, 55 °C for 30 s, and 72 °C for 30 s; followed by 95 °C for 60 s, 55 °C for 30 s, and a final incubation at 95 °C for 30 s. PCR products were electrophoresed in 1% agarose gels.

### Western blot analysis

HCC specimens were homogenized in RIPA buffer (Solarbio Life Sciences, Beijing, China) containing protease inhibitors (Roche). After removing the debris, supernatants were boiled and mixed with an equal volume of 20% glycerol containing 0.02% bromophenol blue. Proteins were separated on 12% SDS-PAGE gels and transferred onto PVDF membranes (Millipore). Membranes were blocked with 5% skim milk in TBST and incubated with primary antibodies in TBST containing 0.5% skim milk overnight at 4 °C. The membrane was treated with primary antibodies against ZCCHC13 (1:500, Abcam), NME2 (1:1000, Abcam), c-MYC (1:1000, Abcam), AKT (1:500, Sangon Biotech), p(Ser129)-AKT1 (1:500, Sangon Biotech), ERK1/2 (1:500, Sangon Biotech), p(Thr202/Tyr204)-ERK1/2 (1:500, Sangon Biotech), CDK1 (1:500, Sangon Biotech), CDK4 (1:500, Sangon Biotech), and GAPDH (1:1000, GeneTex). A horseradish peroxidase-conjugated goat anti-rabbit or anti-mouse immunoglobulin G antibody (1:5000, Sigma) was used as the secondary antibody, and immunoreactive bands were visualized using enhanced chemiluminescence (Amersham Pharmacia Biotech). Densitometry analysis were performed using ImageJ software.

### Immunohistochemistry

HCC samples were fixed overnight with 4% buffered formalin before embedding in paraffin. Paraffin sections were cut at a thickness of 5 µm and dried for 24 h. After dewaxing and rehydration, sections were boiled in citrate buffer for 30 min for antigen retrieval. Then, sections were cooled in citrate buffer, rehydrated with PBS and incubated with 3% H_2_O_2_ for 10 min to block endogenous peroxidase activity. Nonspecific binding sites were blocked with 10% bovine serum albumin (BSA) for 30 min. Sections were stained with the ZCCHC13 antibody (1:100) at 4 °C overnight. Meanwhile, sections incubated with IgG were used as negative controls. After the incubation, sections were washed three times with PBS and incubated with a horseradish peroxidase-conjugated secondary antibody (Jackson ImmunoResearch) for 1 h at room temperature. Peroxidase activity was visualized with a 0.05% solution of the chromogen 3,3′-diaminobenzidine (Sigma) before counterstaining with a haematoxylin solution. Finally, slides were observed under an Olympus BX43 microscope and analyzed with Image-Pro Plus 6.0 software (Media Cybernetics).

### Recombinant protein generation

The full-length ZCCHC13 cDNA (Gene ID: 389874) was cloned into the prokaryotic expression vector pET28a (+) to construct the recombinant expression vector. The recombinant ZCCHC13 protein fused with 6-His tag was produced in a genetically manipulated strain of *E. coli* (BL21) after induction with 0.1 mM IPTG at 25 °C for 24 h. After the purification by Ni^2+^ affinity chromatography, the recombinant ZCCHC13 protein was separated and evaluated by 12% SDS-PAGE gel electrophoresis.

### Seroreactivity analysis

ZCCHC13-specific antibodies were detected in the sera from patients with HCC and healthy individuals using an indirect enzyme-linked immunosorbent assay (ELISA). Briefly, a solution containing 1 µg/mL of the recombinant ZCCHC13 protein (100 µL/well) in coating buffer (Genechem, Shanghai, China) was added to 96-well plates and incubated overnight at 4 °C. Plates were washed thrice with 0.05% Tween 20 and then blocked with 5% BSA (100 µL/well) for 1 h at room temperature. After washing, serum samples (100 µL/well) diluted 1:100 with 3% BSA were added to the plate and incubated for 1 h at room temperature. Diluted goat anti-human IgG labelled with peroxidase (Sigma) was added (100 µL/well) and incubated for 1 h at room temperature. After the incubation, the substrate solution (100 µL/well) was added and incubated for 15 min at room temperature. Subsequently, 3 M H_2_SO_4_ (50 µL/well) was added to each well and the absorbance of each well was read at 450 nm using an MK3 Microplate reader (Thermo). A positive reaction was defined as an absorbance value that exceeded the mean absorbance value of sera from healthy donors (n = 38) by 3 SD. The negative control was established by adding the 6-His fusion protein expressed in *E. coli* from an empty pET28a (+) vector.

### Confocal microscopy

Huh7 cells were grown on coverslips and then fixed with 5% paraformaldehyde at 4 °C for 30 min. After washes with 50 mM NH4Cl, cells were permeabilized with 0.1% Triton X-100 for 15 min at room temperature. Nonspecific binding sites were blocked by 5% BSA for 1 h, and then were incubated with the primary ZCCHC13 antibody overnight. The Texas red-conjugated secondary antibody (ZsBio, Beijing, China) was added and incubated for 1 h at room temperature. After the incubation, mounting medium containing DAPI (ZsBio, Beijing, China) was applied to visualize the nuclei. Immunofluorescence staining was examined with a Carl Zeiss LSM5 EXITER laser scanning confocal microscope (Zeiss, Jena, Germany).

### Methylation-specific PCR (MSP)

Genomic DNA was extracted using a DNeasy System (Qiagen) according to the manufacturer’s instructions. DNA (0.5 g) was treated with bisulfite from the EZ DNA methylation kit (Zymo Research) and then used as the template in the MSP experiments. Sequences of the methylation-specific primers were 5′-TAAAGATTGTAAGGATTTTAAACGA-3′ (forward) and 5′-ATCGATAACACTTAACCTAAACGC-3′ (reverse); sequences of the nonmethylation-specific primers were 5′-TTAAAGATTGTAAGGATTTTAAATGA-3′ (forward) and 5′-ATCAATAACACTTAACCTAAACACA-3′ (reverse). PCR conditions were described in our previous study [16]. Methylated (M) and unmethylated (U) amplification products were detected on 1.2% agarose gels.

### Bisulfite sequencing PCR (BSP)

The methylation status of the ZCCHC13 promoter was detected using BSP. BSP primers were designed using MethyPrimer. The primer sequences were: 5′-ATATTTGTTATAATTGTGGGAGAAG-3′ (forward) and 5′-CAAAAATAACATTTCTACTCTTTCTAAC-3′ (reverse). PCR amplification of bisulfite-treated DNA was performed as previously described [16]. The PCR product was cloned into the pMD18-T vector (Takara). Ten positive clones were randomly selected for sequencing. The sequencing result were visualized using BIQ Analyzer software. A lollipop diagram was generated to show the methylation status of each CpG site.

### ZCCHC13 plasmid construction and transfection

The ZCCHC13 coding region containing *Eco*RI and *Kpn*I restriction sites was amplified using the primers: 5′-CGGAATTCATGAGCAGTAAGGATTTCTTCGC-3′ (forward) and 5′-GGGGTACCGCCTGGGACATTCCTTGGCTAGAT-3′ (reverse). PCR conditions were described in our previous study [16]. The amplification product was cloned into the eukaryotic expression vector pcDNA3.1 (Invitrogen) and confirmed by sequencing. Plasmid transfections were performed using the liposome reagent Fugene 6 (Roche Molecular Biochemicals), according to the manufacturer’s instructions.

### Cell cycle assay

For cell cycle analysis, transfected cells were seeded onto a 12-well plate and cultured for 24 h. The cells were harvested and fixed with 70% alcohol overnight at 4 °C. Subsequently, the cells were washed with PBS and treated with 1% RNase (w/v) at 37 °C for 30 min. Cells were stained with propidium iodide (PI) (250 µg/µL) in the dark for 10 min and analyzed using a flow cytometer (Guava Technologies, Burlingame, CA). The percentages of cells in G0/G1 phase, S phase, and G2/M phase were evaluated by ModFit software (BD, Franklin, NJ, USA).

### Statistical analysis

Student’s t-test (two-tailed), one-way ANOVA and the Mann–Whitney test were used to analyze the data with Prism 6 software (GraphPad Software, San Diego, CA). Differences were considered statistically significant at p < 0.05.

## Results

### ZCCHC13 is expressed at high levels in HCC cells and tissues

**Fig. 1:**
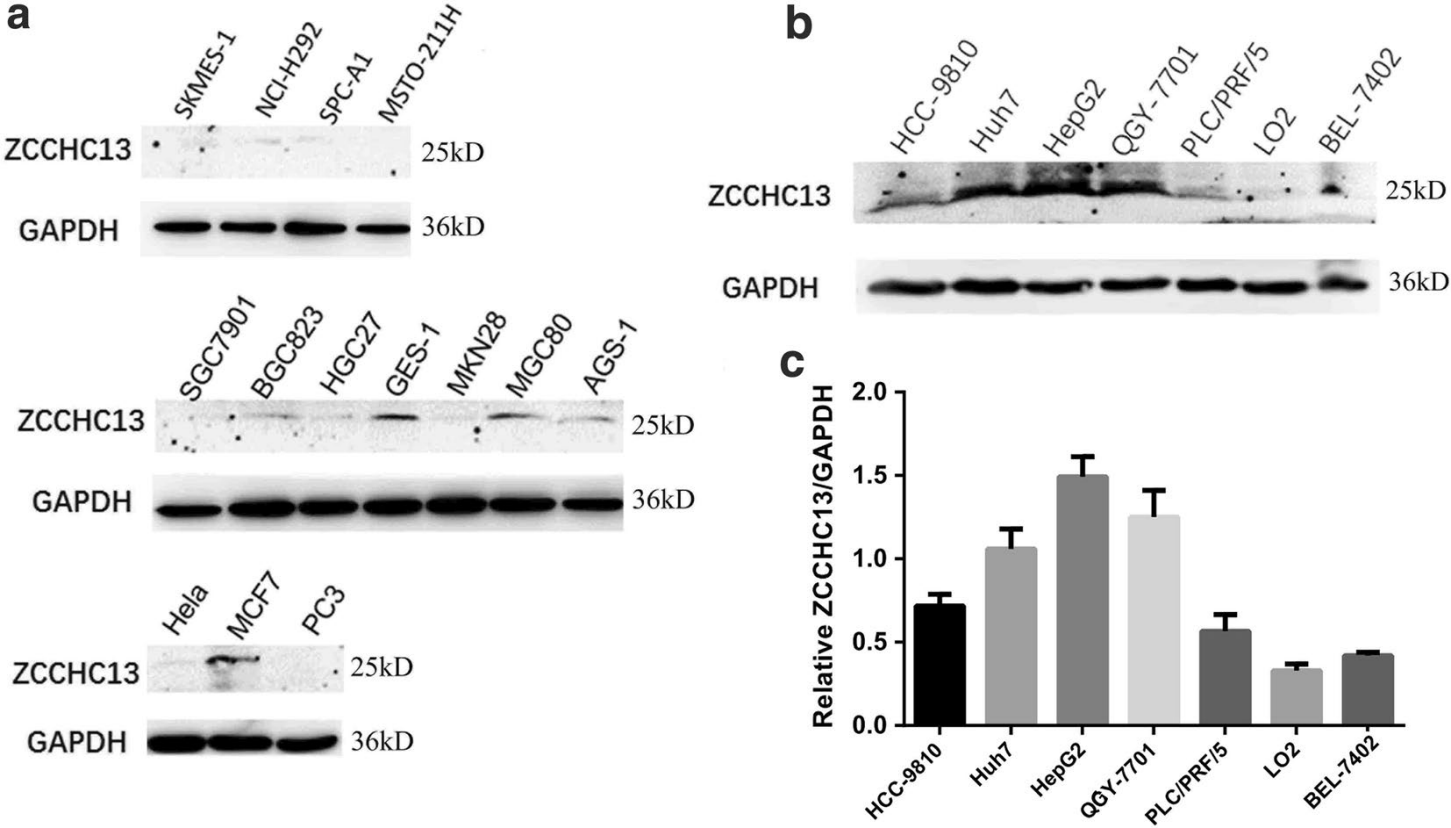
ZCCHC13 expression pattern in multiple human cancer cell lines. **a** Levels of the ZCCHC13 protein in 4 human lung cancer cell lines (SK-MES-1, NCI-H292, SPC-A1, and MSTO-211H), 7 gastric cancer cell lines (SCG7901, BGC823, HGC27, GES-1, MKN28, MGC80, and AGS-1), a breast cancer cell line (MCF7), a cervical cancer cell line (HeLa), and a prostate cancer cell line (PC3). **b** Levels of the ZCCHC13 protein in 7 human liver cell lines, including six HCC cell lines (Huh7, QGY-7701, PLC/PRF/5, HCC-9810, HepG2, and BEL-7402) and one immortalized normal liver cell line (LO2). **c** Quantification of levels of the ZCCHC13 protein in the human liver cancer cell lines. The data are presented as the mean ± SD, n = 3

To analyze the relationship between ZCCHC13 expression and carcinogenesis, we detected the levels of the ZCCHC13 protein in a series of different human cancer cell lines, including lung, stomach, breast, cervical, and prostate cancer cell lines. Western blot results revealed varying levels of the ZCCHC13 protein in cancer cells, ranging from clearly detectable to almost undetectable (Fig. 1a). Notably, strong immunoreactivity was observed in more aggressive human liver cancer cell lines, including Huh7, QGY-7701, PLC/PRF/5, HCC-9810, HepG2, and BEL-7402, in particular the highest levels were observed in HepG2, Huh7 and QGY-7701 cells (Fig. 1b). However, the level of the ZCCHC13 protein was almost undetectable in the immortalized normal liver cell line LO2. The intensity of densitometric imaging also showed remarkably higher levels of the ZCCHC13 protein in aggressive liver cancer cells compared with LO2 cells (Fig. 1c). The reliability of the ZCCHC13 antibody used for Western blotting was validated in cells subjected to RNA interference (Additional file 1: Figure S1a).

**Fig. 2:**
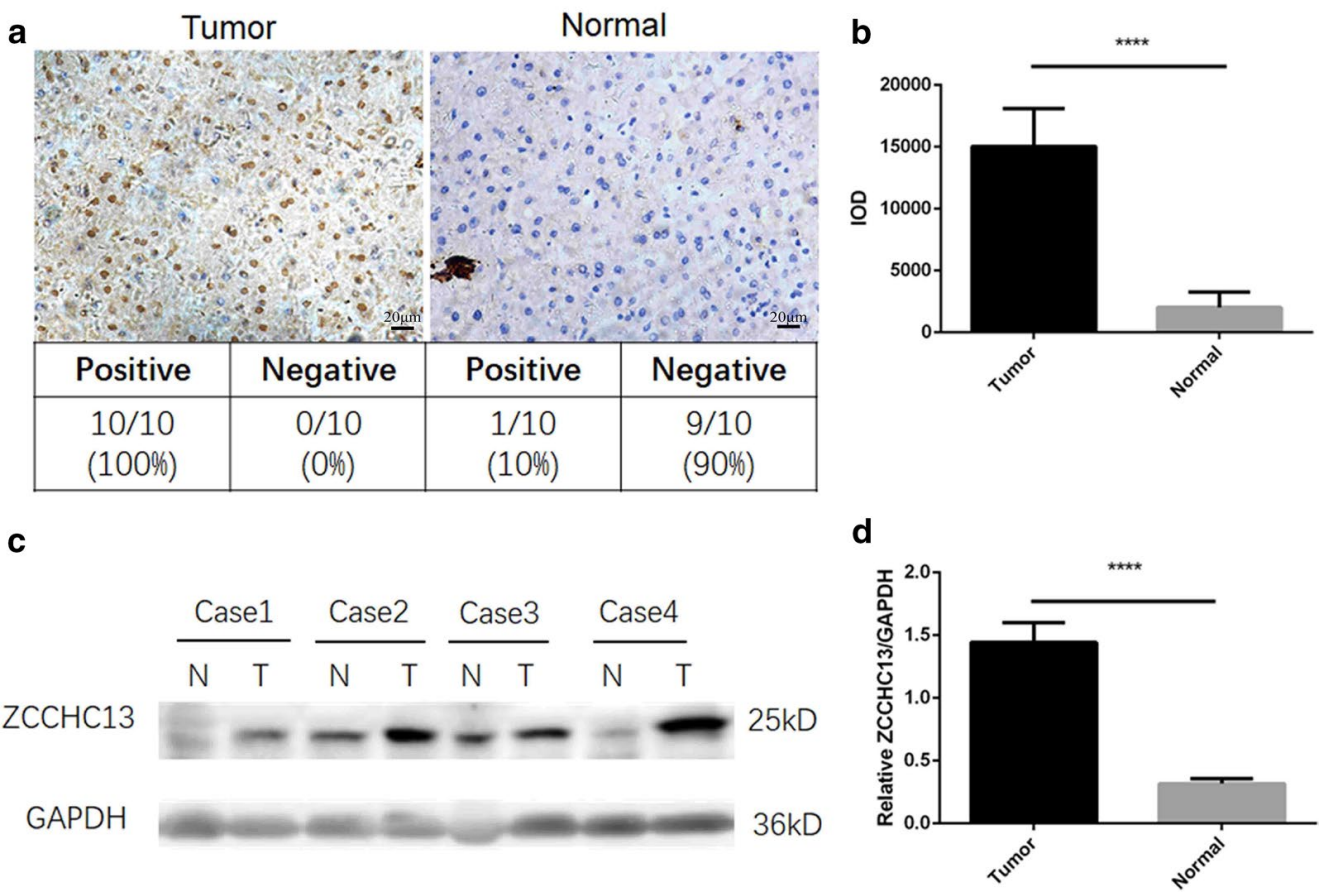
ZCCHC13 expression pattern in HCC specimens. **a** ZCCHC13 immunoreactivity was detected using immunochemistry at a high magnification of ×400. Scale bar, 20 μm. **b** Western blot showing the levels of the ZCCHC13 protein in 4 independent samples; T, tumor tissue; N, normal tissue. **c** Statistical analysis of the mean IOD for ZCCHC13-positive staining in tissue sections according to the following formula: mean IOD = IOD/area of the tumor section. Three images from each slide were assessed. The results are presented as the mean ± SD of three images from each slide. **d** Quantification of Western blot results for the ZCCHC13 protein in HCC specimens (****p < 0.0001)

**Fig. 3:**
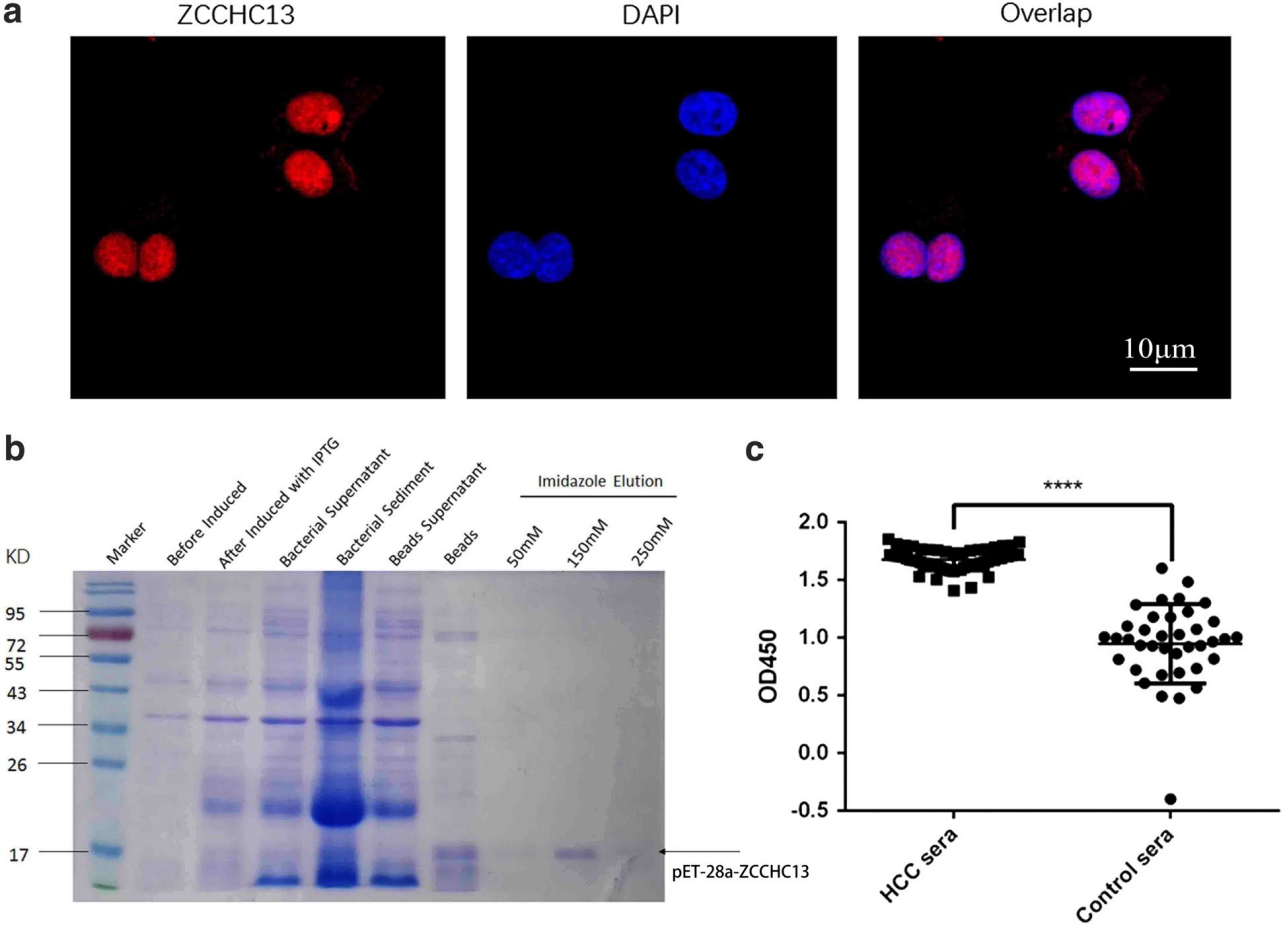
Analysis of the ZCCHC13 subcellular localization and ZCCHC13-specific antibodies in human sera. **a** The endogenous ZCCHC13 protein was distributed in the nucleus of Huh7 cells. Merged images showed the co-localization between the ZCCHC13 protein and nuclei stained with DAPI. **b** The recombinant ZCCHC13 protein was expressed at the predicted molecular size of approximately 18 kD, as particularly evident in the lane for the 150 mM imidazole elution buffer. **c** Responses of serum from patients with HCC and healthy donors to ZCCHC13, as determined using an ELISA (****p < 0.0001)

In view of the emerging roles of ZCCHC13 in human liver cancers, we examined the expression of the ZCCHC13 protein in ten pairs of paraffin-embedded HCC specimens using immunohistochemistry. Positive signals for ZCCHC13 staining were detected in all tumor tissues. However, ZCCHC13 immunoreactivity was not detected in adjacent nontumor samples, except for only one case (Fig. 2a). A statistical analysis of all immunochemical images indicated significantly higher ZCCHC13 immunoreactivity in tumors than in normal tissues (Fig. 2b). In addition to the immunohistochemistry analysis, Western blotting was performed to confirm the levels of the ZCCHC13 protein in HCC specimens. Immunoblots revealed that ZCCHC13 expression was frequently upregulated in 78.6% (48/61) of tumor tissues compared with 5% (3/60) of nontumor tissues. The levels of the ZCCHC13 protein were increased in HCC tumor tissues compared to nontumor tissues (Fig. 2c, Additional file 1: Figure S1b). The quantitative analysis revealed significantly higher levels of the ZCCHC13 protein in tumor tissues than in adjacent nontumor tissues (Fig. 2d). To confirm the positive immunohistochemical staining in the nucleus of tissue sections, confocal microscopy was used to detect the cellular localization of ZCCHC13. Immunofluorescence staining revealed that the ZCCHC13 protein was predominately located in the nucleus of Huh7 cells (Fig. 3a).

### The ZCCHC13 antibody is present at high levels in the sera of patients with HCC

With the aim of comparing the levels of the immunogenic ZCCHC13 antibody in patients with HCC and healthy individuals, the full-length recombinant ZCCHC13 protein was produced, separated on SDS-PAGE gels, and stained with Coomassie blue. A single band was observed in the expected position with a molecular weight of approximately 18 kD (Fig. 3b). After immunoblot validation of the isolated recombinant protein, an ELISA was established using the recombinant ZCCHC13 protein. According to the results of the seroreactivity analysis, the ZCCHC13 antibody present in the sera of patients with HCC was highly reactive to the recombinant ZCCHC13 protein. The mean absorbance value (1.68 ± 0.01) recorded for patients with HCC (n = 61) was significantly higher than the mean value (0.95 ± 0.05) for healthy controls (n = 38) (p < 0.0001) (Fig. 3c), indicating that ZCCHC13 protein was strongly recognized by sera from patients with HCC compared with healthy controls. More importantly, the ZCCHC13 protein might be used as an immunogenic tumor antigen in cancer therapy.

### The ZCCHC13 promoter is hypomethylated in HCC cells

**Fig. 4:**
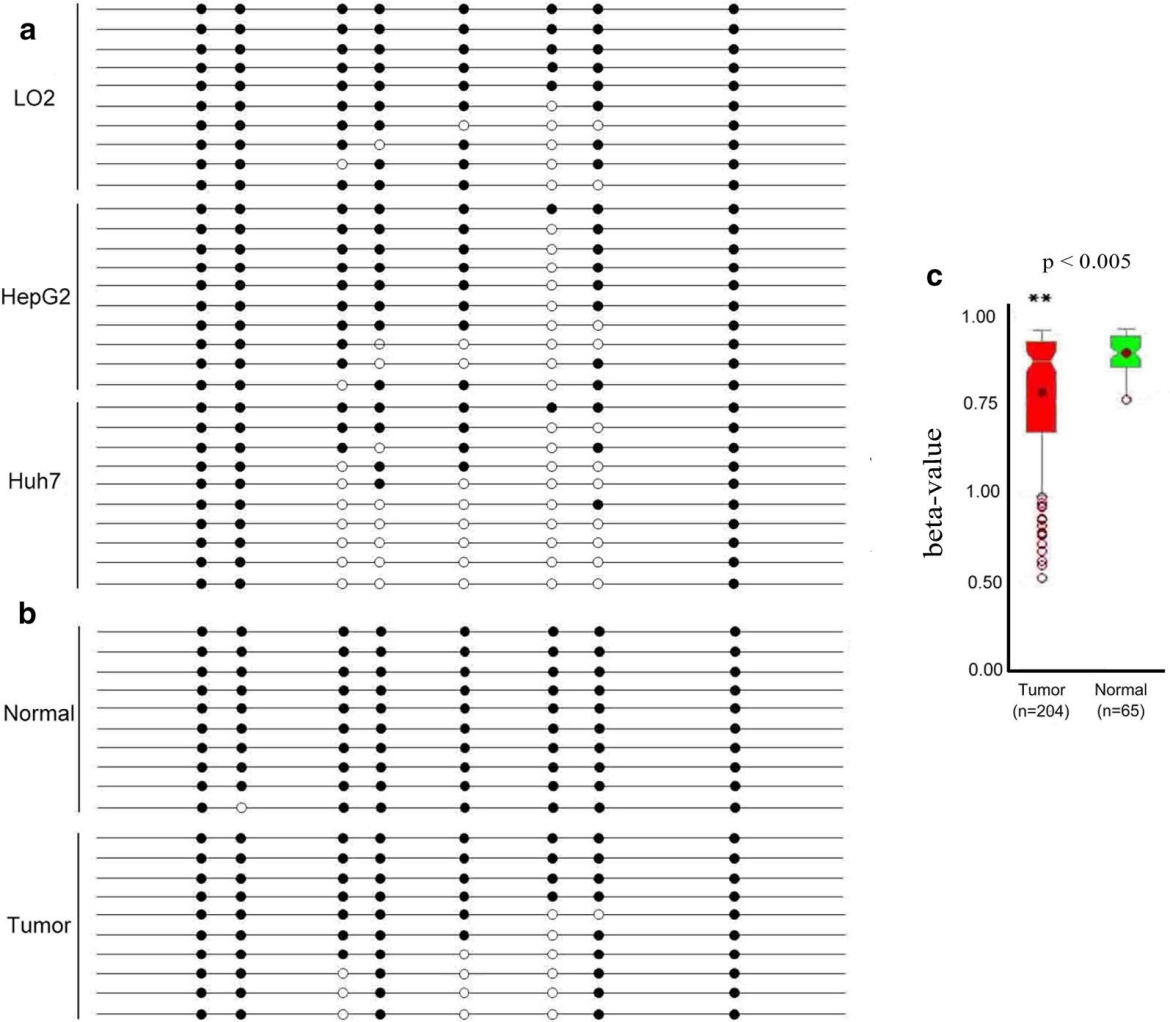
DNA methylation status of ZCCHC13 in human liver cancer cell lines and specimens. **a** Methylation status of 8 CpG dinucleotides in the ZCCHC13 promoter in LO2, HepG2, and Huh7 cells. Columns indicate alterations in the methylation of CpG sites contained in the BSP amplicon based on 10 cloned sequences for each cell line. **b** ZCCHC13 methylation status in 10 pairs of tumor and normal samples. Black dot, methylated CG; white dot, unmethylated CG. **c** Methylation data were obtained from TCGA through the MethHC database for pairs of tumor (n = 204) and (n = 65) normal tissues. Beta values range from 0 to 1, with 0 representing the unmethylated DNA and 1 representing the completely methylated DNA

A DNA methylation analysis was performed using BSP to investigate the underlying mechanisms of ZCCHC13 overexpression in HCC cell and tissues. Based on the sequencing results, five of eight CpG sites within the ZCCHC13 promoter were frequently hypomethylated in HepG2 and Huh7 cells; conversely, these sites were hypermethylated in LO2 cells (Fig. 4a). Furthermore, 10 tumor tissues and 10 matched nontumor tissues were collected to validate the ZCCHC13 promoter methylation status in HCC tissue sections. Similar to the trends observed in cell lines, the ZCCHC13 promoter was significantly hypomethylated in tumor tissues compared with normal tissues (Fig. 4b). The methylation level of the ZCCHC13 promoter was further analyzed in HCC samples using cohorts from the MethHC dataset (http://methhc.mbc.nctu.edu.tw/php/index.php). The average beta values for the ZCCHC13 promoter in tumor samples (n = 204) were much lower than in matched normal samples (n = 65) (p < 0.005) (Fig. 4c). Although the methylation data from TCGA showed a similar result (Additional file 1: Figure S2a), the expression of the ZCCHC13 mRNA was not significantly altered in tumors and normal tissues from the TCGA (Additional file 1: Fig S2b, c) and ICGC databases (Additional file 1: Table S3). DNA methylation and gene expression data were analyzed with Wanderer [17] and HCCDB [18] tools.

### 5-Aza-induced demethylation upregulates ZCCHC13 expression

**Fig. 5:**
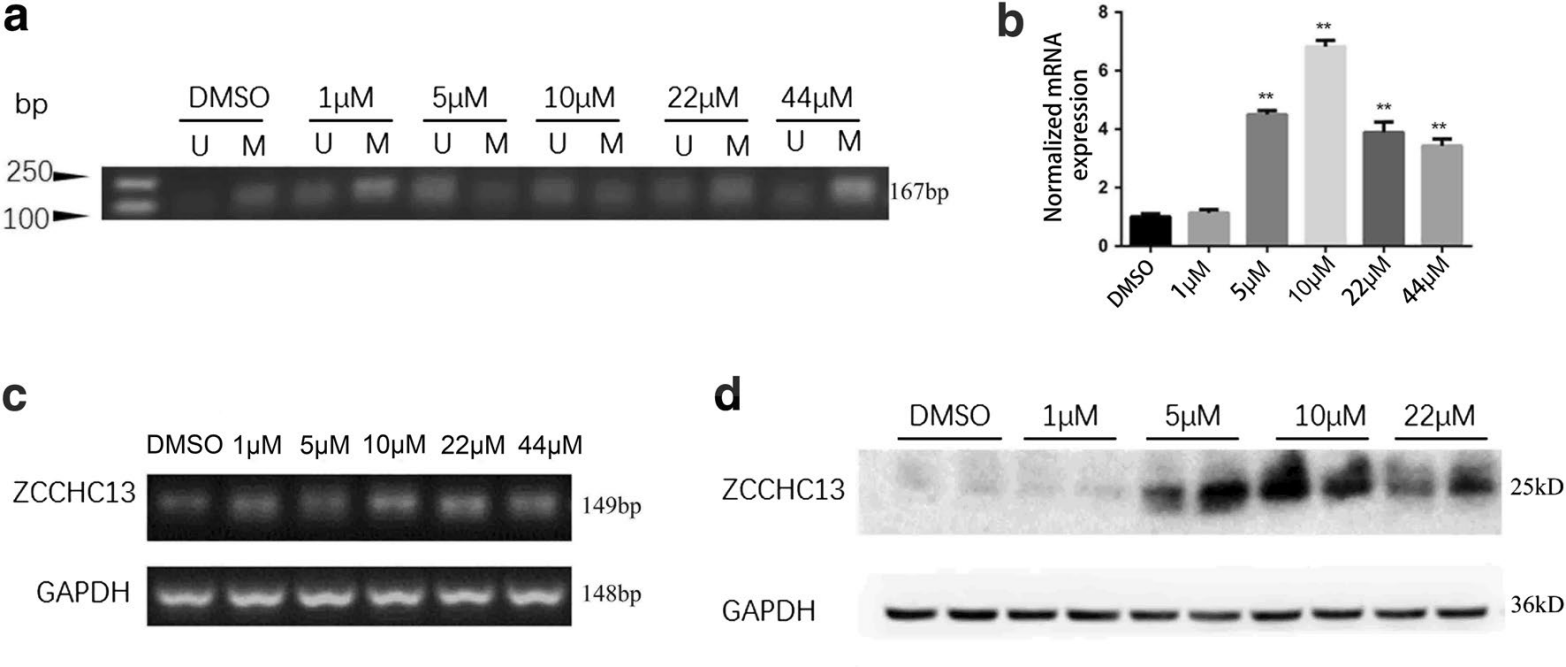
5-Aza-induced demethylation increases ZCCHC13 expression in human liver cancer cells. **a** Results of MSP, **b** RT-qPCR, **c** RT-PCR and **d** Western blot analysis showing that 5-Aza attenuated the methylation of the ZCCHC13 promoter in HepG2 cells. U: Unmethylated MSP amplicons; M: methylated MSP amplicons. GAPDH was used as an internal control. The results are presented as the mean ± SD of three independent experiments (*p < 0.05 and **p < 0.01)

To evaluate the changes in ZCCHC13 expression caused by hypomethylation, HepG2 cells were treated with the demethylating agent 5-Aza. MSP results revealed significantly increased levels of unmethylated amplification products of ZCCHC13 in 5-Aza-treated groups compared with DMSO controls (Fig. 5a). To further confirm the effect of demethylation effect on the mRNA level, RT-qPCR was performed in HepG2 cells treated with five different concentrations of 5-Aza (1, 5, 10, 22, or 44 µM). The RT-qPCR results showed that the relative expression of the ZCCHC13 mRNA was increased in a dose-dependent manner, with significant effects observed in cells treated with 1, 5, and 10 µM 5-Aza (p < 0.01) (Fig. 5b). Semi-quantitative RT-PCR data supported the specificity of the amplification product and the results of RT-qPCR (Fig. 5c). Although a slight decrease in the expression of ZCCHC13 mRNA was detected in cells treated with 22 and 44 µM 5-Aza, the increase and differences were still statistically significant compared with DMSO controls (p < 0.01). Western blot analysis confirmed the significant increase in the levels of the ZCCHC13 protein following the 5-Aza treatment. A dose-dependent increase in the levels of the ZCCHC13 protein was observed in cells treated with 1, 5, and 10 µM concentrations of 5-Aza (Fig. 5d). These results indicated that 5-Aza-induced demethylation remarkably increased the levels of the ZCCHC13 mRNA and protein ZCCHC13, with the maximum level observed in cells treated with 10 µM 5-Aza.

### ZCCHC13 overexpression promotes HCC cell growth

**Fig. 6:**
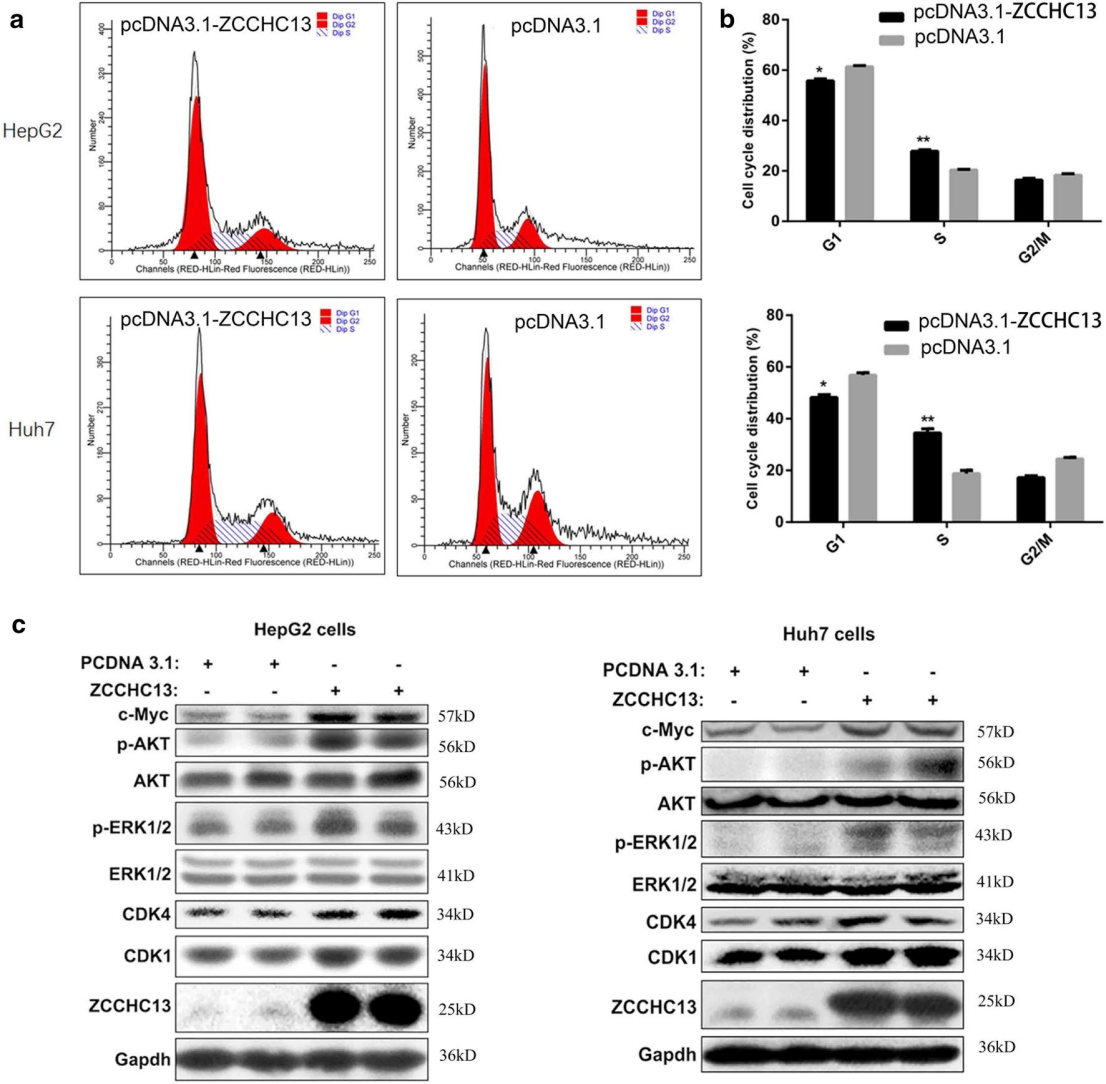
Overexpression of ZCCHC13 promotes the G1-S transition and regulatory gene expressions in liver cancer cells. **a** Representative profiles of a flow cytometry analysis are shown in the left panel. **b** The percentages of cells in various phases of the cell cycle were calculated and presented as the mean ± SD from three experiments in the right panel (*p < 0.05, **p < 0.01). **c** Western blot analysis showing increased levels of the CDK1, CDK4 and c-MYC proteins and the phosphorylated AKT and ERK proteins following ZCCHC13 overexpression

To further elucidate the oncogenic role of ZCCHC13 in carcinogenesis, HepG2 and Huh7 cells were transfected with a recombinant ZCCHC13 vector or empty vector. Then, we examined the effect of ZCCHC13 overexpression on the cell cycle distribution. The flow cytometry analysis revealed an enhancement of cell cycle progression in cells overexpressing ZCCHC13 compared with cells transfected with the empty vector (Fig. 6a). Upon overexpression of the ZCCHC13 protein, the percentage of cells in G0/G1 phase was significantly decreased, while the percentage of cells in S phase was significantly increased (Fig. 6b). Based on these results, the effect of ZCCHC13 on cell growth was associated with facilitating the G1-S cell transition.

We speculate that the overexpression of ZCCHC13 in human liver cells may lead to the abnormal induction of various signaling pathways. For this purpose, we detected the alterations in the levels of critical regulators of the cell cycle and related signaling pathways in HepG2 and Huh7 cells. As shown in the Western blots, levels of the CDK1 and CDK4 protein were increased in HepG2 and Huh7 cells overexpressing the ZCCHC13 protein. This increase was accompanied by increased phosphorylation of AKT1 (Ser129) and ERK1/2 (Thr202/Tyr204), as well as increased expression of c-MYC (Fig. 6c). Taken together, overexpression of ZCCHC13 promotes cell cycle progression.

## Discussion

The biological role of ZCCHC13 in cancers remains unknown, although it is expressed in the human testis. Here, we presented a comprehensive study of ZCCHC13 in human liver cancer. Western blot analysis of multiple human cancer cell lines revealed that the normal liver cell line LO2 expressed the ZCCHC13 protein at much lower levels than common liver cancer cells. Clinicopathological examinations showed a similar trend, confirming that ZCCHC13 expression was downregulated in normal liver tissues. Based on these results, ZCCHC13 functions as an oncogene during the carcinogenesis of human liver cancer. As expected, sera from patients with HCC displayed a significantly stronger response to the recombinant ZCCHC13 protein than sera from healthy controls, verifying the increased levels of the ZCCHC13 protein in tissues from patients HCC. Moreover, ZCCHC13 overexpression was attributed to hypomethylation of the ZCCHC13 promoter.

Aberrant hypomethylation of CpG islands within promoter regions leads to transcriptional activation in cancer cells [19, 20]. According to a recent study by Barbara of HCC, promoter hypomethylation is widely distributed in many oncogenes, suggesting a critical role for gene hypomethylation in carcinogenesis [19]. Of the genes identified in our previous study, we speculate that ZCCHC13 expression may be influenced by DNA methylation in human cancers. In present study, we discovered that five CpG sites in the ZCCHC13 promoter region were significantly hypomethylated in human liver cancer cells and tissues. Decreased hypomethylation levels coincided with increased expression of ZCCHC13. Liver cancer cells treated with the demethylating agent 5-Aza exhibited a dose-dependent increase in ZCCHC13 expression at both the mRNA and protein levels. Thus, hypomethylation of ZCCHC13 is likely an epigenetic factor involved in the carcinogenesis of HCC. This finding is consistent with the well-established concept that promoter methylation is normally associated with gene silencing.

**Fig. 7:**
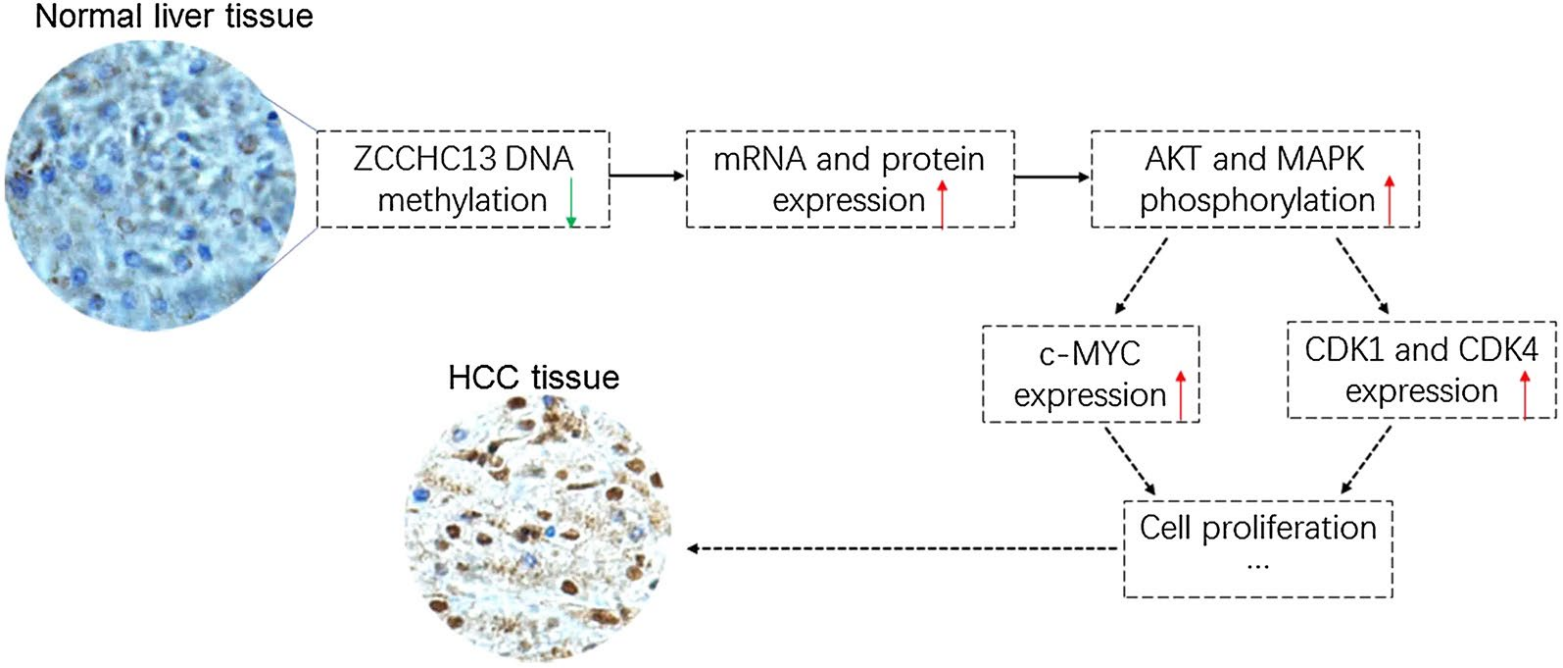
The working model of ZCCHC13 in HCC carcinogenesis. ZCCHC13 promoter hypomethylation increases the levels of its mRNA and protein in normal liver tissues. Then, ZCCHC13 overexpression aberrantly stimulates the activation of the PI3K/AKT and MAPK signaling pathways, upregulating the expression of the downstream component c-MYC. Finally, cell cycle progression is enhanced through the G1 and G2 checkpoints, which speed entry into mitosis in the presence of the c-MYC-linked kinases CDK1 and CDK4

The ZCCHC13 protein contains four C-X_2_-C-X_4_-H-X_4_-C (CCHC) boxes, which are highly conserved in eukaryotes. CCHC-type zinc finger proteins have been shown to be involved in different biological processes, including nucleic acid binding, protein–protein interactions, and transcriptional activation, among others [21]. A large-scale systematic resequencing analysis revealed a potential correlation between the truncated variant of ZCCHC13 and X-linked mental retardation [22]. However, the function of ZCCHC13 in cancer has yet to be determined. In the present study, we used hepatocellular carcinoma as a model to delineate the biological role of ZCCHC13 in cancers. As summarized in our working model (Fig. 7), DNA demethylation during carcinogenesis upregulates ZCCHC13 expression, resulting in increased cell growth from normal cells to cancerous cells. The AKT/ERK/c-MYC signaling pathway played an important role in the ZCCHC13-evoked increase in tumor cell growth. Although the exact mechanism requires further validation, our results collectively support the hypothesis that DNA hypomethylation is the initial epigenetic abnormality in human cancers.

## Conclusions

In summary, our study revealed that ZCCHC13 is expressed at high levels in HCC tumor tissues and is closely associated with DNA hypomethylation of the promoter region. We also found that ZCCHC13 promotes cell proliferation through cell cycle-linked signaling pathways. ZCCHC13 is a novel molecule acting in the AKT/ERK/c-MYC pathway. This study may provide a potential novel therapeutic target for the diagnosis and treatment of HCC.

## Additional file

**Additional file 1.** Additional tables and figures.

## Abbreviations

ZCCHC13: zinc-finger CCHC-type containing 13
HCC: hepatocellular carcinoma
NOA: non-obstructive azoospermia
CT: cancer–testis
AFP: alpha-fetoprotein
5-Aza: 5-Aza-2′-deoxycitidine
PI: propidium iodide
BSA: bovine serum albumin
CCHC: C-X_2_-C-X_4_-H-X_4_-C
MSP: methylation-specific PCR
BSP: bisulfite sequencing PCR
qRT-PCR: quantitative reverse transcription PCR
