# Supplementary material for: ZCCHC13-mediated induction of human liver cancer is associated with the modulation of DNA methylation and the AKT/ERK signaling pathway
Source: J Transl Med. 2019 Apr 2;17:108. doi: 10.1186/s12967-019-1852-0 (PMC6444591; doi:10.1186/s12967-019-1852-0)
Supplement: Supplementary file 1 — Additional file 1. Additional tables and figures. [file 12967_2019_1852_MOESM1_ESM.docx]

Additional Material for

**ZCCHC13-mediated induction of human liver cancer is associated with the modulation of DNA methylation and the AKT/ERK signalling pathway**

Zhiming Li^1,2,3*^, Zhi Li^2^, Linjun Wang^2^, Chen Long^2^, Zaozao Zheng^2^, Xuan Zhuang^3*^

| **Table S1. Patients’ baseline characteristics (N = 61)** | | |
| --- | --- | --- |
| **Characteristic** | **No.** | **%** |
| **Age, years** |  |  |
| Median | 56 | - |
| Range | 32-82 | - |
| >65 | 25 | 41% |
| ≤65 | 36 | 59% |
| **Sex** |  |  |
| Male | 30 | 49% |
| Female | 31 | 51% |
| **ECOG performance status** |  |  |
| 0 | 35 | 57% |
| 1 | 26 | 43% |
| **Child-Pugh status** |  |  |
| A | 47 | 77% |
| B | 14 | 23% |
| **AFP>ULN** |  |  |
| Yes | 23 | 38% |
| No | 38 | 62% |
| **Positive hepatitis status** |  |  |
| Hepatitis B | 12 | 20% |
| Hepatitis C | 18 | 30% |
| **Disease stage at study entry (TNM classification)** | |  |
| IIIA/IIIB | 24 | 39% |
| IV | 37 | 61% |
| **Grading (AJCC) at initial diagnosis** |  |  |
| Well-differentiated | 21 | 34% |
| Moderately well-differentiated | 26 | 43% |
| Poorly differentiated | 14 | 23% |
| Abbreviations: AFP, alpha-fetoprotein; AJCC, American Joint Committee on Cancer; ULN, upper limit of normal; ECOG, Eastern Cooperative Oncology Group. | | |
|  | | |

| **Table S2. Summary table of specimen number in the biological tests** | | | | |
| --- | --- | --- | --- | --- |
| **Samples** | **BSP (DNA)** | **IHC (tissue)** | **WB (protein)** | **ELISA (serum)** |
| **HCC individuals** |  |  |  |  |
| HCC tissues | 10 | 10 | 61 | - |
| Adjacent tissues | 10 | 10 | 60 | - |
| Blood sera |  |  |  | 61 |
| **Healthy individuals** | |  |  |  |
| Blood sera | - | - | - | 38 |
| Abbreviations: BSP, bisulfite sequencing PCR; IHC, immunohistochemistry; WB, Western Blot; ELISA, enzyme-linked immunosorbent assay. | | | | |

| **Table S3. Gene expression summary of ZCCHC13** | | | | | | | | |
| --- | --- | --- | --- | --- | --- | --- | --- | --- |
| **Dataset** | **Platform** | **Source** | **P-value** | **Type** | **Nums** | **Mean** | **STD** | **IQR** |
| **HCCDB1** | Rosetta/Merck Human RSTA Custom Aﬀymetrix 1.0 microarray | GSE22058 | 0.00315 | HCC | 100 | 4.626 | 0.2096 | 0.2871 |
|  |  |  |  | Adjacent | 97 | 4.717 | 0.2184 | 0.3064 |
| **HCCDB3** | Rosetta/Merck Human RSTA Aﬀymetrix 1.0 microarray | GSE25097 | 0.02248 | HCC | 268 | 0.04114 | 0.008186 | 0.007 |
|  |  |  |  | Adjacent | 243 | 0.04265 | 0.006732 | 0.008 |
|  |  |  |  | Cirrhotic | 40 | 0.0385 | 0.006349 | 0.0065 |
|  |  |  |  | Healthy | 6 | 0.03967 | 0.004633 | 0.003 |
| **HCCDB4** | Illumina HumanHT-12 V4.0 expression beadchip | GSE36376 | 0.5973 | HCC | 240 | 5.762 | 0.1261 | 0.1602 |
|  |  |  |  | Adjacent | 193 | 5.755 | 0.1318 | 0.1624 |
| **HCCDB11** | Illumina Human Whole-Genome DASL HT | GSE46444 | 0.03869 | HCC | 88 | 7.485 | 1.484 | 2.78 |
|  |  |  |  | Adjacent | 48 | 8.193 | 2.056 | 3.761 |
| **HCCDB12** | Agilent-014850 Whole Human Genome | GSE54236 | 0.9512 | HCC | 81 | 1.954 | 0.7773 | 1.04 |
|  |  |  |  | Adjacent | 80 | 1.962 | 0.7033 | 0.8635 |
| **HCCDB13** | Aﬀymetrix Human Genome U219 Array | GSE63898 | 0.03926 | HCC | 228 | 4.095 | 0.1691 | 0.2077 |
|  |  |  |  | Adjacent | 168 | 4.135 | 0.2031 | 0.2245 |
| **HCCDB15** | RNA-Seq | **TCGA-LIHC** | 0.05634 | HCC | 351 | 0.00749 | 0.07331 | 0 |
|  |  |  |  | Adjacent | 49 | 0 | 0 | 0 |
| **HCCDB16** | Aﬀymetrix Human Gene 1.0 ST Array | GSE64041 | 0.9638 | HCC | 60 | 5.236 | 0.2371 | 0.2549 |
|  |  |  |  | Adjacent | 60 | 5.238 | 0.2324 | 0.3242 |
| **HCCDB17** | Illumina HumanHT-12 V4.0 expression beadchip | GSE76427 | 0.2381 | HCC | 115 | 6.484 | 0.1159 | 0.165 |
|  |  |  |  | Adjacent | 52 | 6.508 | 0.1252 | 0.14 |
| **HCCDB18** | RNA-Seq | **ICGC-LIRI-JP** | 0.1745 | HCC | 212 | 0.00066 | 0.007057 | 0 |
|  |  |  |  | Adjacent | 177 | 0 | 0 | 0 |

Abbreviations: Nums, numbers; STD, standard deviation; IQR: interquartile range.

**Figure S1**


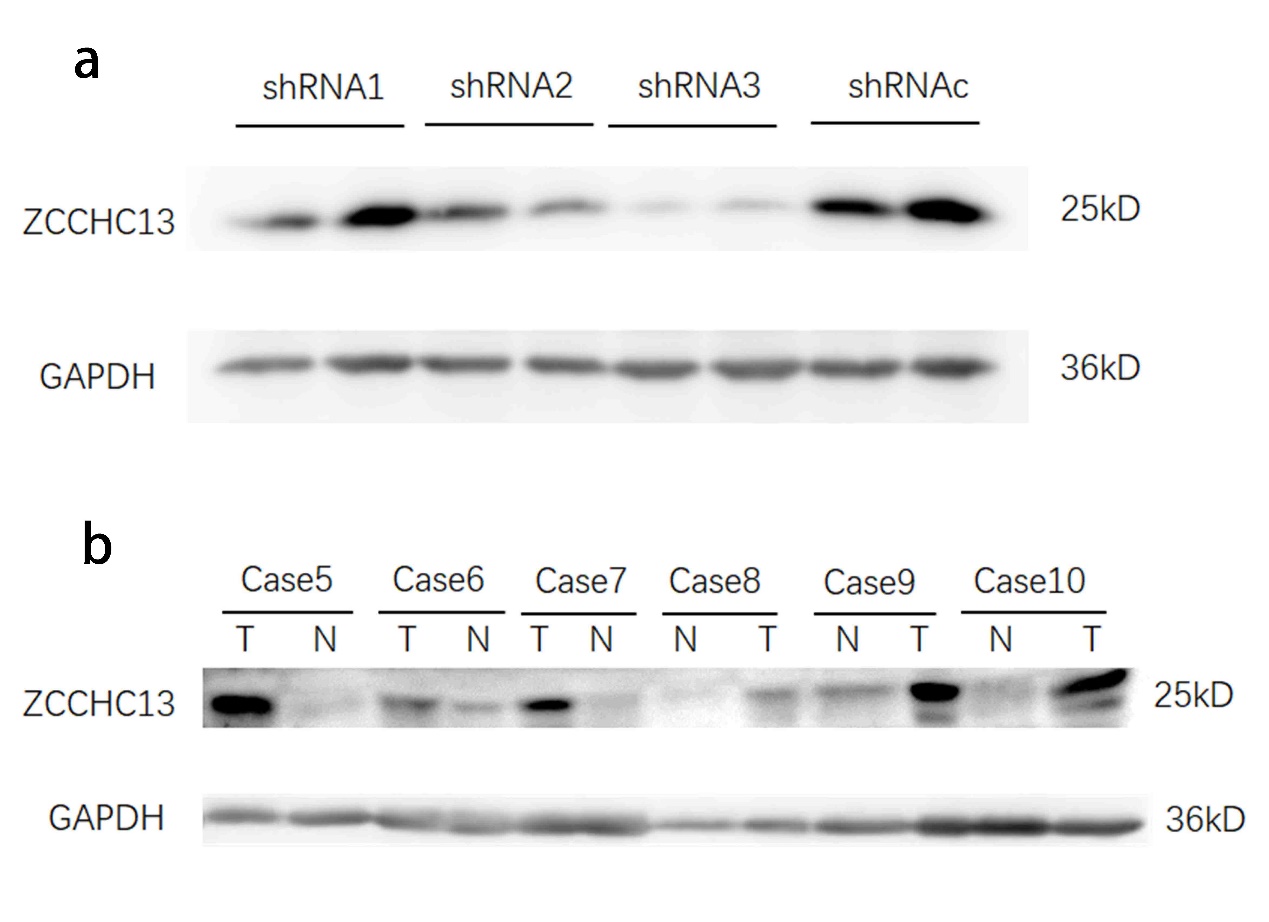


**Figure S1 ZCCHC13 antibody spcificity detection and expression profiles in the HCC tissues.**

(a) Antibody specificity was assessed by measuring the relevant signal in Huh7 cells by RNA interference. (b) Expression levels of ZCCHC13 protein in 6 HCC independent tissues were analyzed by Western blot; T, Tumor; N, Normal.

**Figure S2**


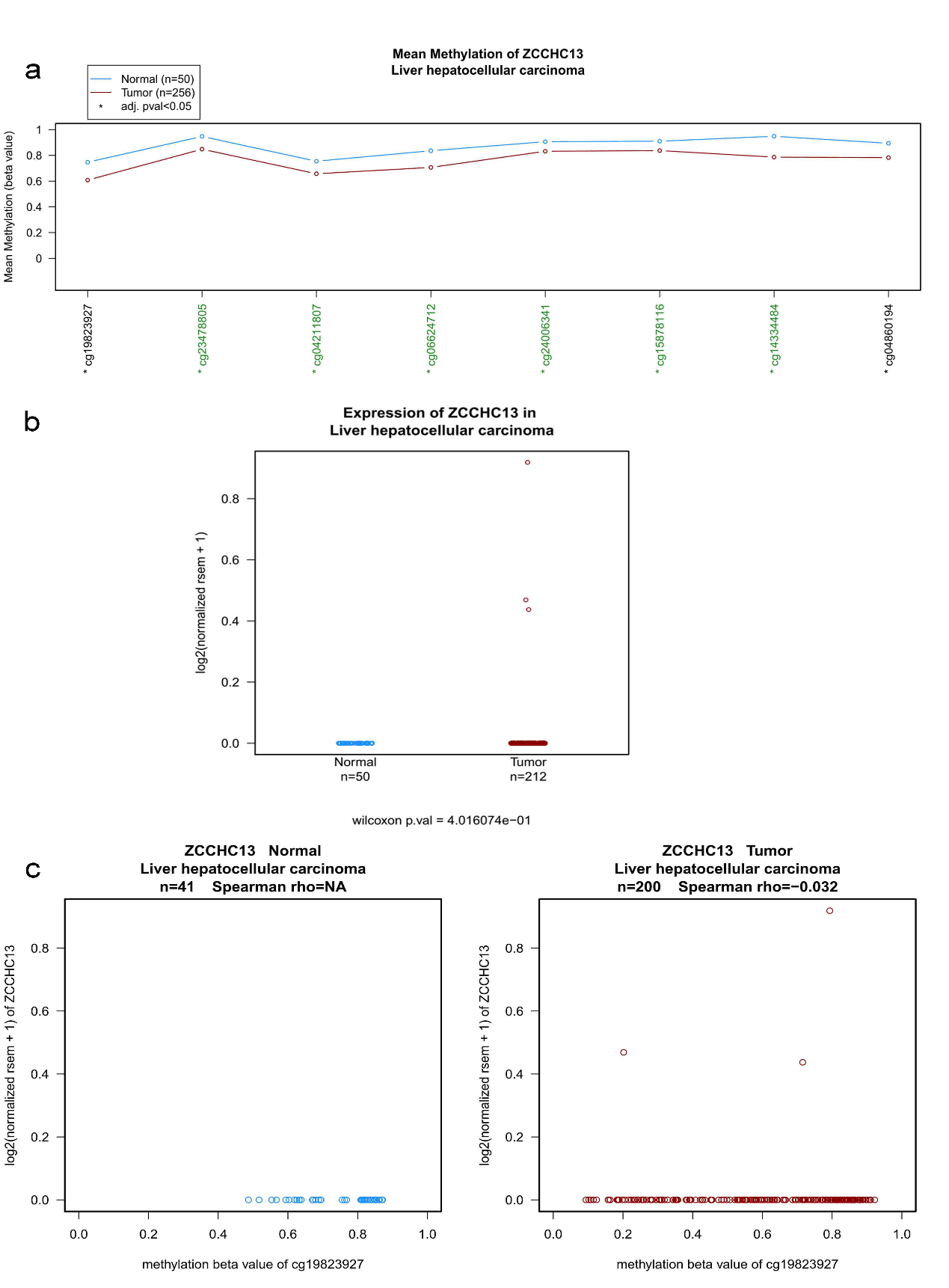


**Figure S2. Methylation and gene expression analysis of ZCCHC13 in normal and liver hepatocellular carcinoma tissues from** **Wanderer viewer.**

(a) Profile plots displaying the beta values for each probe in normal (upper panel, blue marks) and tumour tissue samples (bottom panel, red marks) from the TCGA-LIHC dataset. Lines link different probes corresponding to the same sample. The CpGs showing statistically significant differences are highlighted with an asterisk (Wilcoxon adjusted p-value). Probes located in CpG islands are coloured in green. (b) Boxplot showing the expression levels in all the normal (blue) and tumour samples (red) from the TCGA-LIHC dataset. The expression values are log2-transformed normalized RSEM values. (c) Plots showing the correlation between DNA methylation beta values and RNAseq expression for the normal (left panel, blue) and tumour tissue samples (right panel, red). The correlations were obtained by calculating Spearman’s rho.
